# Comprehensive Assessment of Reflux Burden and Mucosal Integrity in Children: Insights from MNBI and Impedance–pH Monitoring

**DOI:** 10.3390/children12111486

**Published:** 2025-11-03

**Authors:** Felicia Galos, Alexandra Ilie, Mihai Daniel Luca Mirea, Raluca Teodora Radulescu, Mara Ioana Ionescu

**Affiliations:** 1 Division of Pediatrics, Carol Davila University of Medicine and Pharmacy, 050474 Bucharest, Romania; felicia.galos@umfcd.ro; 2 Department of Pediatrics II, Marie Curie Emergency Children’s Hospital, 077120 Bucharest, Romania; alexandra.ilie0125@rez.umfcd.ro (A.I.); mihai-daniel-luca.mirea@rez.umfcd.ro (M.D.L.M.); raluca-teodora.radulescu@rez.umfcd.ro (R.T.R.); 3 Division of Physiology—Neuroscience, Carol Davila University of Medicine and Pharmacy, 02001 Bucharest, Romania

**Keywords:** pediatric gastroesophageal reflux disease, multichannel intraluminal impedance-pH monitoring, mean nocturnal baseline impedance, acid exposure time, acid reflux, gas reflux

## Abstract

**Highlights:**

**What are the main findings?**

- Mean nocturnal baseline impedance (MNBI) correlates inversely with acid exposure and effectively identifies esophageal mucosal impairment in children with GERD, particularly in those ≥1 year old.
- Distinct age-related reflux patterns were observed: infants exhibited more weakly acidic and liquid reflux episodes, while older children showed a predominance of gas reflux.

**What is the implication of the main finding?**

- MNBI provides an objective, reproducible marker that complements standard pH-impedance metrics and improves diagnostic accuracy in pediatric GERD.
- Age-specific MNBI thresholds are needed to optimize diagnosis and guide management, as adult cut-offs are not directly applicable to children.

**Abstract:**

Background: Pediatric gastroesophageal reflux disease (GERD) presents with heterogeneous phenotypes across ages, and diagnostic challenges persist. Multichannel intraluminal impedance and pH (MII-pH) monitoring is the current gold standard, while mean nocturnal baseline impedance (MNBI) has emerged as a marker of mucosal integrity in adults. Pediatric normative data are still lacking. We aim to characterize age-related reflux patterns and assess the association between MNBI and pathological acid exposure in children undergoing MII-pH monitoring. Methods: We retrospectively analyzed 226 children evaluated with 24 h MII-pH monitoring between 2017 and 2025. Clinical and laboratory data were reviewed. Children were stratified by age (<1 year vs. ≥1 year). Pathological reflux was defined as reflux index (RI) > 10% in infants and >7% in older children. MNBI was measured at distal channels (Z5, Z6) during nocturnal recumbency. Correlations between MNBI and RI were assessed. Diagnostic performance of MNBI for pathological acid exposure was evaluated using ROC analysis. Results: Infants had more total reflux episodes, particularly weakly acidic and liquid, whereas older children exhibited more gas reflux. MNBI correlated inversely with RI (Z6 r = −0.337; Z5 r = −0.281; both *p* < 0.0001). In infants, MNBI poorly discriminated pathological acid exposure. In older children, MNBI showed better performance, with optimal cut-offs of ~2525 Ω (Z6) and 3079 Ω (Z5) yielding specificities > 85%. Conclusions: MNBI is a reproducible, age-sensitive marker of reflux burden in children, best suited for older children where it complements RI in diagnosing acid-mediated GERD. Larger prospective studies are needed to establish pediatric reference values.

## 1. Introduction

Gastroesophageal reflux disease (GERD) is defined as the occurrence of troublesome symptoms and/or complications resulting from the retrograde movement of gastric contents into the esophagus, distinguishing it from physiological gastroesophageal reflux (GER), which is typically self-limiting and asymptomatic in healthy individuals [1,2]. GERD is a common condition in the pediatric population, with its prevalence and clinical manifestations varying considerably across different age groups. Reported prevalence rates may reach up to 38%, depending on the population studied and diagnostic criteria used [3]. In infants, GERD may present with regurgitation, irritability, feeding difficulties, or poor weight gain, whereas in older children and adolescents, typical symptoms include heartburn, epigastric discomfort, dysphagia, or respiratory complaints such as chronic cough or wheezing [4,5].

Diagnosing GERD in children remains challenging due to the nonspecific nature of symptoms and the absence of a universally accepted diagnostic gold standard. Among the available tools, combined multichannel intraluminal impedance and pH monitoring (MII-pH) has emerged as the most comprehensive and informative method for evaluating reflux. This technique allows for the detection and characterization of both acid and non-acid reflux events, their temporal correlation with symptoms, their physical properties (liquid, gas, or mixed), and the proximal extent of each event.

Studies have also highlighted the clinical relevance of non-acid and gas reflux in children, especially in relation to extra-esophageal symptoms and possible associations with atopy [6,7]. Despite increasing use of MII-pH in clinical practice, data on its diagnostic yield and correlation with clinical and immunologic features in pediatric populations remain limited.

Mean nocturnal baseline impedance (MNBI) has emerged as a valuable and promising parameter for assessing esophageal mucosal integrity in both adults and children [8,9,10,11]. Unlike conventional reflux metrics that quantify the frequency or acidity of reflux episodes, MNBI reflects the baseline electrical resistance of the esophageal lumen, which is inversely correlated with mucosal permeability and damage. Lower MNBI values are indicative of impaired mucosal integrity and are often associated with increased acid exposure or chronic reflux-related inflammation [12,13]. As such, MNBI provides complementary information to standard pH-impedance parameters and is increasingly recognized as a surrogate marker of cumulative reflux burden and mucosal injury. In adults, an MNBI threshold below 1500 Ω is commonly considered indicative of pathological reflux [14,15]. However, in the pediatric population, normative reference values are lacking, and age-specific thresholds remain undefined. Baseline impedance values can differ substantially across age groups due to physiological, anatomical, and technical factors. In infants, frequent liquid feeds, prolonged supine positioning, and smaller esophageal diameter may contribute to lower impedance compared with older children and adults, while maturation of esophageal structure and function leads to more stable values with age [14,16,17]. These differences underscore the need for establishing age-specific normative MNBI thresholds in pediatric populations. Through this study, we aim to contribute to the characterization of MNBI patterns in children with suspected GERD and to explore its potential diagnostic value in identifying pathologic acid exposure, thereby supporting the development of pediatric-specific reference standards.

Additionally, this study aims to retrospectively evaluate the diagnostic performance of MII-pH monitoring in children with suspected GERD and to characterize age-related differences in reflux patterns and MNBI values in order to assess its potential role as a diagnostic marker of esophageal mucosal integrity in children with suspected gastroesophageal reflux disease. By characterizing reflux patterns in a large pediatric cohort, we aim to contribute to a more nuanced understanding of GERD in children and highlight the added value of impedance-based assessment in clinical decision-making.

## 2. Materials and Methods

### 2.1. Study Design

We conducted a retrospective, single-center, observational study at the Department of Pediatrics, Marie Curie Emergency Children’s Hospital, Bucharest, Romania. The study was designed to evaluate reflux patterns and their clinical correlations in pediatric patients who underwent MII-pH monitoring for suspected GERD. The study period extended from January 2017 to June 2025. Ethical approval for the study was obtained from the Institutional Ethics Committee (37332/07.08.2025), and the study was conducted in accordance with the principles outlined in the Declaration of Helsinki. Written informed consent was obtained from the caregivers for the performance of MII-pH monitoring, the confirmation chest radiograph for catheter positioning, and the use of anonymized data for inclusion in this retrospective analysis.

### 2.2. Study Population and Eligibility Criteria

Pediatric patients aged from infancy to adolescence who underwent 24 h MII-pH monitoring were screened for inclusion. Patients were included if the MII-pH study was technically complete (>18 h of recording) and interpretable, and if comprehensive clinical data were available in the medical records. Exclusion criteria were established to minimize confounding factors and included known neurological disorders, congenital malformations of the gastrointestinal tract, previous anti-reflux surgical interventions, or incomplete study recordings (<18 h). With regard to medication use, children receiving ongoing anti-reflux therapy at the time of monitoring were not included unless acid-suppressive or prokinetic agents had been temporarily discontinued prior to the study for at least 7 days, when clinically appropriate. This ensured that all included patients underwent MII-pH evaluation in the absence of active pharmacologic interference with gastric acid secretion or gastrointestinal motility.

### 2.3. Data Collection and Variables

Data were collected by reviewing hospital medical records and laboratory investigations. Demographic data included patient age, sex, and anthropometric measurements such as weight percentiles. Clinical history focused on the presence of allergic comorbidities, including asthma, eczema, allergic rhinitis, and documented food allergies. Laboratory parameters such as serum hemoglobin, immunoglobulin A (IgA), immunoglobulin E (IgE), and vitamin D levels were recorded, where available, to assess possible associations between immunologic markers and reflux characteristics.

### 2.4. MII-pH Monitoring Procedure

**Figure 1:**
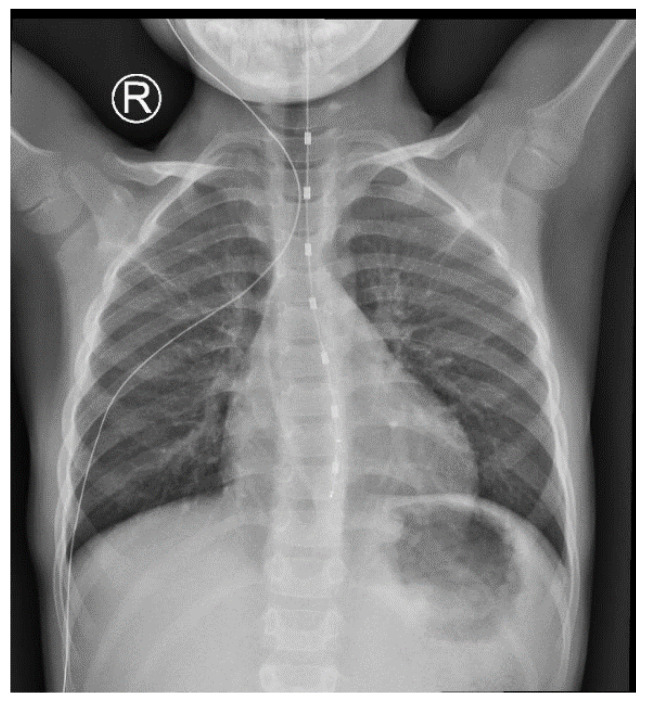
Chest radiograph from one of our patients for verification of esophageal impedance–pH catheter position. The catheter is visualized along the esophagus with electrodes in appropriate position. Lung fields and mediastinal structures appear within normal limits. The R sign indicated the right side of the patient.

All patients underwent 24 h MII-pH monitoring using the Digitrapper ™ pH-Z Testing System (Medtronic, Watford, UK), with impedance catheters selected according to patient body length: infant (<75 cm), pediatric (75–150 cm), and adult (>150 cm) models [18]. Each catheter was equipped with seven impedance channels and a single distal esophageal pH sensor. pH sensors were calibrated before use, following the manufacturer’s specifications. Catheters were inserted transnasally, and the estimated insertion depth was calculated using the Strobel formula (0.252 × body length in cm + 5) [11,19]. This formula, however, tends to overestimate esophageal length in taller children, as it is derived from infant data. To ensure correct sensor positioning, catheter placement was verified by plain chest radiography before initiating the recording (Figure 1).

During the 24 h monitoring period, patients were advised to follow their usual feeding patterns and daily activities. Medications known to affect gastric acid secretion or gastrointestinal motility were withheld when clinically appropriate. Caregivers were instructed to maintain a detailed diary documenting the child’s symptoms, body posture, food intake, and sleep–wake cycles. Data were collected using the portable recording unit and subsequently analyzed with specialized software provided by the manufacturer (AccuView Reflux Software, Medtronic, Watford, UK). Each study was manually reviewed and interpreted by a pediatric gastroenterologist with expertise in MII-pH analysis to ensure accurate reflux event identification and classification.

**Figure 2:**
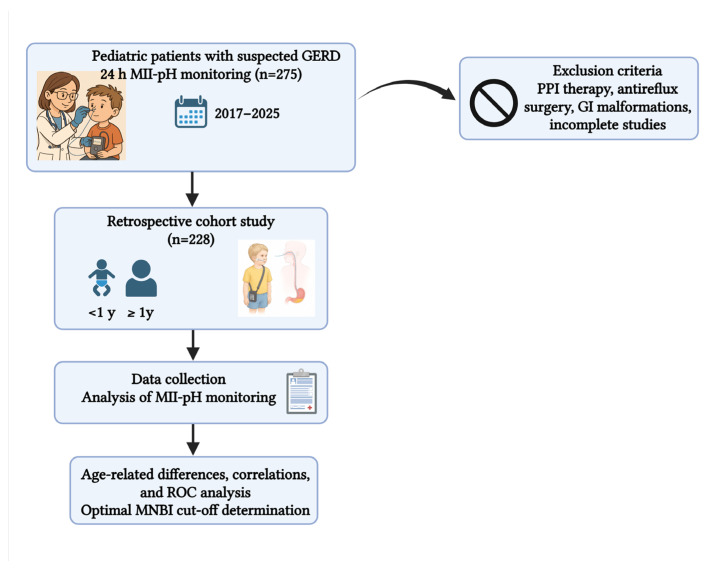
Flowchart of the study design. Pediatric patients with suspected GERD undergoing 24 h MII-pH monitoring (2017–2025) were screened. After exclusions, 228 patients were analyzed by age group, with MII-pH data assessed for age-related differences, correlations, and ROC analysis to determine the optimal MNBI cut-off. Created in Biorender, Ionescu, M.I.I. (2025).

The study design and patient selection are illustrated in Figure 2.

### 2.5. Definitions and Diagnostic Criteria

Reflux events were classified based on standard MII-pH definitions. Liquid reflux episodes were defined as a retrograde decrease in impedance to less than 50% of the baseline value, detected in at least two consecutive distal impedance channels. Acid reflux was defined as a retrograde bolus movement detected by impedance sensors associated with a fall in distal esophageal pH below 4. Non-acid reflux events were identified by impedance-detected bolus movements without a pH drop. Gas reflux was defined by a rapid increase in impedance exceeding 3000 Ω across at least two consecutive sensors, with one site having more than 7000 Ω, indicative of intraluminal gas. Mixed reflux events involved both liquid and gas components [11].

GERD diagnosis was based on established pediatric thresholds. A reflux index (RI), also known as acid exposure time (AET), representing the percentage of time the esophageal pH remained below 4, greater than 7% was considered diagnostic of acid GERD in children above one year of age, while in infants an AET above 10% was considered abnormal. RI values below 4% were considered normal, while values between 4% and 7% were classified as borderline or inconclusive [11,18,20]. An abnormal number of total reflux episodes, defined as more than 100 episodes in infants (under one year of age) or more than 70 in older children, was also considered diagnostic [11,18]. Gas-only reflux was classified as pathological when more than approximately 70 episodes were recorded during the 24 h study. This threshold reflects our institutional practice, derived from an average of cut-off values reported in previous studies [21].

The Boix-Ochoa score was also used to interpret the results for GER. Developed specifically for children, a Boix-Ochoa score below 11.99 typically indicates a normal, non-pathological level of acid reflux, while higher scores suggest a higher likelihood of GERD in children [22,23].

MNBI was retrospectively calculated for all patients using the two most distal impedance channels (Z5 and Z6) during the supine (recumbent) period of nocturnal sleep. MNBI was determined by averaging three artifact-free, stable 10 min intervals centered around 1:00 a.m., 2:00 a.m., and 3:00 a.m., in accordance with the methodology described by Martinucci et al. [8]. Periods containing pH drops, swallows, or reflux events were excluded to ensure baseline signal integrity. Due to technical limitations of our pH-MII system, automated or simplified MNBI calculation across the entire nighttime period, as reported in other protocols, was not feasible [17]. All measurements were manually reviewed and validated.

### 2.6. Statistical Analysis

All data were entered and analyzed using GraphPad Prism 9.00 (GraphPad Software Inc., Boston, USA). Descriptive statistics were used to summarize patient demographics, clinical characteristics, and laboratory findings. Continuous variables were expressed as mean ± standard error of the mean (SEM) or median with interquartile range (IQR), depending on data distribution. Categorical variables were presented as frequencies and percentages. Student’s *t*-test or Mann–Whitney U test was applied to compare continuous variables between groups, depending on the distribution as determined by normality tests. Depending on the distribution of the data, either Pearson’s correlation coefficient (for normally distributed variables) or Spearman’s rank correlation coefficient (for non-parametric data) was used to assess the strength and direction of associations between continuous variables. To evaluate the diagnostic performance of selected parameters, receiver operating characteristic (ROC) curve analysis was performed. The area under the ROC curve (AUC) was used to determine the sensitivity and specificity of these variables in discriminating between patients with and without pathological reflux. A *p*-value of less than 0.05 was considered statistically significant.

## 3. Results

### 3.1. Study Population

A total of 275 patients were initially enrolled in the study. After excluding those receiving proton pump inhibitor therapy, patients with incomplete MII-pH recordings, and individuals with a history of fundoplication or congenital malformations of the gastrointestinal tract, 226 patients were retained for the final analysis. The cohort was balanced, with 50.88% (115/226) males. The mean age was 5.77 ± 0.33 years (range 0.08–17.76 years). 48 patients were <1 year and 178 were ≥1 year. The distribution of infants and older children in our cohort reflects the clinical population referred for reflux evaluation at our tertiary center rather than a predefined sampling strategy, thus representing a real-world convenience sample of patients undergoing MII-pH monitoring.

Children were referred for MII-pH monitoring due to a wide spectrum of symptoms suggestive of GERD. The most frequent indications included vomiting and regurgitation, followed by abdominal or epigastric pain, chronic cough, and feeding difficulties such as feeding refusal or growth retardation. Other reported manifestations included crying, irritability, wheezing, apnea, eructation, dysphagia, nausea, heartburn, and laryngitis. These presentations reflect both typical and extraesophageal symptoms of GERD across the pediatric age spectrum, encompassing infants with regurgitation and feeding-related issues as well as older children with abdominal or respiratory complaints.

The patient’s demographics, reflux characteristics, and laboratory findings are illustrated in Table 1.

### 3.2. Patient Characteristics

The study cohort was stratified by age into infants (<1 year) and older children (≥1 year). The mean age of the infants was 0.52 ± 0.03 years (range: 0.08–0.95 years), while that of the older group was 7.18 ± 0.35 years (range: 1.01–17.76 years, *p* < 0.0001). Weight percentiles were similar between groups, with median values of 22.24 (IQR: 4.25–38.99) in infants and 22.33 (IQR: 4.66–56.25) in older children (*p* = 0.382).

### 3.3. pH Findings

Median RI was slightly higher in infants (3.15%, IQR: 1.2–8.75) than in older children (2.45%, IQR: 1.1–5.12, *p* = 0.196), though with a wider range in both groups. Among infants, a pathological RI was observed in 16.7% of cases (8/48), whereas in older children, pathological RI was present in 18.5% of cases (33/178), demonstrating a similar prevalence between the two groups. The number of acid reflux episodes was comparable, with a median of 53 (IQR: 28.25–120.5) in infants and 56 (IQR: 29.75–81.5, *p* = 0.552) in older children. Long acid reflux episodes (>5 min) were infrequent in both groups, with medians of 1 and 0, respectively (*p* = 0.197). Boix-Ochoa scores showed slightly higher medians in infants (17.9) compared to older children (16.45, *p* = 0.196).

### 3.4. Impedance-Detected Episodes

Infants had a higher number of total impedance-detected episodes (62.5, IQR: 42.5–92.75) compared with older children (46.5, IQR: 26.75–67.25, *p* = 0.0002). Abnormal numbers of impedance-detected reflux episodes were identified in 22.9% of infants (11/48) and in 23.6% of older children (42/178), indicating a comparable prevalence across age groups. Infants exhibited a higher frequency of weakly acid reflux episodes (29, IQR 16–47.5 vs. 13.5, IQR 6.75–25; *p* < 0.0001) and liquid reflux episodes (51.5, IQR 29.5–76.5 vs. 27.5, IQR 15–48; *p* < 0.0001). Gas reflux episodes were more frequent in older children (49.5, IQR 24.75–94.25) compared to infants (32.5, IQR 24–49.75; *p* = 0.035). Esophageal extension was significantly greater in infants (median 78.6%) than in older children (median 69.5%; *p* = 0.013). Mixed and non-acid reflux episodes were rare and did not differ significantly between groups. Esophageal extension was generally higher in infants (median 78.6%) compared with older children (69.5%, *p* = 0.013).

In the infant group, two patients exhibited both a pathological RI and an abnormal number of impedance-detected episodes, whereas in the older children group, this overlap was observed in 15 patients.

### 3.5. Symptom Association

Symptom association indices were higher in infants, with 45.16% showing a positive symptom index (SI ≥ 50%) compared with 27.35% of older children (*p* = 0.999). The symptom association probability (SAP ≥ 95%) was significantly more frequently positive in infants (57.14%) than in older children (34.95%, *p* = 0.031).

Mean Nocturnal Baseline Impedance (MNBI)

Mean MNBI values were broadly similar between groups. At channel Z6, MNBI was 4354 ± 259.9 Ω in infants and 4344 ± 167.8 Ω in older children (*p* = 0.740). At channel Z5, values were 4610 ± 247.8 Ω in infants and 4719 ± 159.6 Ω in older children (*p* = 0.993). The range was wide in both groups.

Laboratory Findings

Older children demonstrated higher hemoglobin levels (12.82 ± 0.09 g/dL) compared to infants (11.51 ± 0.2 g/dL, *p* < 0.0001). IgE levels tended to be higher in older children (136 ± 34.18 U/mL) than in infants (7.97 ± 1.37 U/mL), but this difference was not statistically significant (*p* = 0.106). IgA concentrations were significantly higher in older children (113.6 ± 7.29 U/mL) compared to infants (59.37 ± 25.4 U/mL; *p* = 0.0007). Vitamin D levels were higher in infants (64.74 ± 7.79 ng/mL) compared to older children (27.31 ± 1.5 ng/mL; *p* < 0.0001). However, many of these values are also age-dependent.

A significant inverse correlation was observed between weight percentile and the number of gas reflux episodes (r = –0.126, R^2^ = 0.016, *p* = 0.039), indicating that lower weight percentiles were associated with increased gas reflux. No other significant correlations were identified.

### 3.6. Correlations Between RI and MNBI

Correlation analysis demonstrated a strong negative relationship between MNBI values at channel Z6 (r = −0.337, R2 = 0.114, *p* < 0.0001), and channel Z5 (r = −0.281, R2 = 0.078, *p* < 0.0001), respectively, and RI. This indicates that as RI increased, MNBI values decreased significantly.

Age-related differences were observed; therefore, the data were analyzed separately by age group.

**Figure 3:**
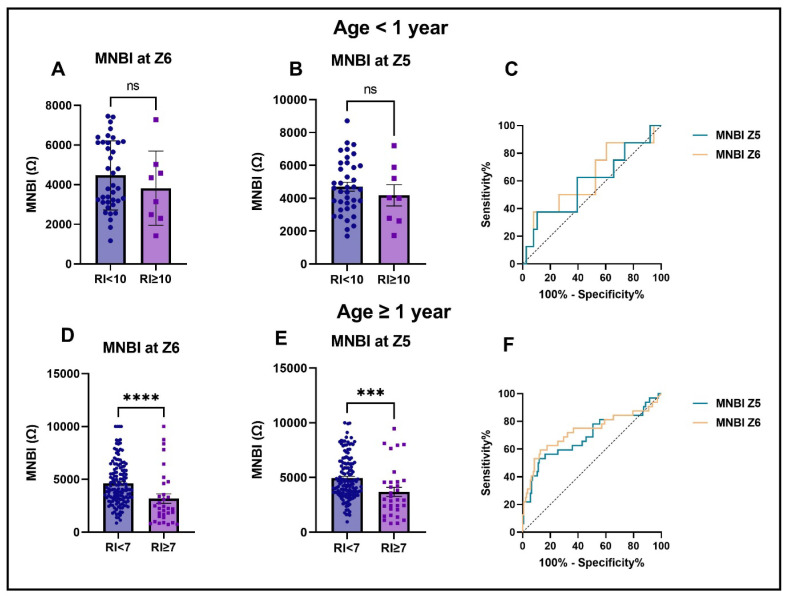
Comparison of MNBI values and diagnostic performance by age group and RI. (**A**,**B**) MNBI values at Z6 and Z5 in infants (<1 year) stratified by RI < 10 vs. RI ≥ 10. No significant difference observed (ns). (**D**,**E**) MNBI values at Z6 and Z5 in older children (≥1 year) stratified by RI < 7 vs. RI ≥ 7. MNBI values were significantly lower in the higher RI group (*** *p* < 0.001, **** *p* < 0.0001). (**C**,**F**) Receiver operating characteristic (ROC) curves for MNBI at Z5 and Z6 for infants and older children, respectively. Abbreviations: ns, not significant.

Infants (<1 year): When using the higher RI threshold for this group (RI > 10%), MNBI demonstrated limited discriminatory ability for diagnosing GERD. No significant differences were found in MNBI values at either Z6 or Z5 between infants with normal and abnormal RI (Figure 3A,B). The diagnostic performance was modest, with an AUC of 0.618 (95% CI: 0.390–0.846, *p* = 0.29) for Z6 and 0.585 (95% CI: 0.351–0.819, *p* = 0.45) for Z5 (Figure 3C).

Older children (≥1 year): Using the established abnormal threshold of RI > 7%, MNBI showed stronger discriminatory capacity. At Z6, the AUC was 0.728 (95% CI: 0.610–0.846, Figure 3F), with an optimal cut-off of 2525 Ω, yielding a sensitivity of 59.4% and specificity of 85.9%. At Z5, the AUC was 0.694 (95% CI: 0.578–0.810, Figure 3F), with an optimal cut-off of 3079 Ω, corresponding to a sensitivity of 53.1% and specificity of 87.3%. MNBI values at both Z6 and Z5 differed significantly between patients with normal and abnormal RI (*p* < 0.0001 and *p* = 0.0005, respectively, Figure 3D,E). These findings suggest that MNBI can serve as an indirect marker of acid-related mucosal damage in pediatric patients.

## 4. Discussion

In this single-center retrospective study of children undergoing 24 h MII-pH for suspected GERD, we characterized age-dependent reflux patterns and evaluated the diagnostic utility of MNBI. Four principal findings emerged: (i) infants and older children displayed distinct impedance phenotypes; (ii) symptom-reflux association differed by age; (iii) MNBI correlated inversely with acid exposure; (iv) MNBI identified pathological reflux in older children but not in infants when age-appropriate RI thresholds were applied.

While current European and North American guidelines recommend MII-pH primarily in children with persistent symptoms despite acid suppression and normal endoscopy [4], our center frequently uses MII-pH as a first-line diagnostic tool. This approach is supported by its minimally invasive nature and ability to give a comprehensive profile of reflux events. Our results support this approach, particularly in older children, where MNBI can add objective information on mucosal integrity to guide clinical decision-making, especially in cases with borderline RI or atypical symptoms [24].

Infants (<1 year) exhibited more total reflux episodes, particularly weakly acidic and liquid events with greater proximal extend, findings consistent with physiology dominated by milk feeds, recumbency, and immature esophageal clearance and with previous literature [25,26]. Older children showed more gas reflux, likely reflecting aerophagia, dietary patterns, or behavioral factors [27]. Despite these differences in event composition and ascent, median RI was not significantly different between groups, underscoring the value of impedance for phenotyping beyond pH metrics alone. Although some differences, such as the higher frequency of weakly acidic reflux in infants and gas reflux in older children, reached statistical significance, these findings should be interpreted with caution due to overlap between groups and the potential for physiological variability.

SI positivity was similar across ages, but SAP was significantly higher in infants. Because reflux events and nonspecific symptoms (e.g., irritability, regurgitation) are both frequent in infancy, temporal coincidence may inflate SAP. Nonetheless, impedance-based temporal analysis adds useful clinical context in early life [28]. Given differences in diary reliability and symptom specificity, SI and SAP should be interpreted alongside objective metrics. These non-significant results may reflect limited statistical power rather than the absence of true clinical differences. Therefore, these observations should be considered exploratory and hypothesis-generating rather than definitive.

Across the cohort, MNBI at both distal channels correlated inversely with RI, supporting MNBI as a marker of impaired epithelial integrity in the presence of higher acid exposure [20]. These findings align with previous adult and pediatric data showing that MNBI reflects cumulative reflux burden rather than isolated events [29]. The similarity of mean MNBI values between infants and older children suggests that disease activity, rather than age, is the main determinant of impedance variability when measurements are standardized to nocturnal, artifact-free periods.

In our study, MNBI was calculated manually by averaging three stable 10 min nocturnal periods free of swallows, reflux episodes, or pH drops. This approach, although not automated, is consistent with the conventional methodology described in pediatric and adult literature and remains a validated means of assessing esophageal mucosal integrity. While newer systems allow automated or “simple” MNBI computation across the entire nocturnal supine period, prior comparative studies have demonstrated strong correlations between conventional and automated methods (r > 0.85 in infants and r > 0.9 in older children), with comparable diagnostic accuracy and discriminatory power for GERD phenotypes [30]. Therefore, although our manual approach may require greater observer input and introduces minor variability related to segment selection, it is unlikely to have affected the reproducibility or validity of our findings. Nonetheless, harmonization of MNBI measurement protocols across centers—particularly through standardized automated algorithms—would further enhance inter-study comparability and reproducibility in future pediatric research.

In infants, MNBI showed limited diagnostic performance (AUC 0.618–0.585, RI > 10%), likely due to high physiologic reflux burden [20], feed-related impedance instability [31], and small esophageal diameter [32]. In contrast, MNBI effectively discriminated pathological reflux in older children (RI > 7%; AUC 0.728 at Z6 and 0.694 at Z5). Optimal cut-offs (~2525 Ω and ~3079 Ω) yielded high specificity (≈86–87%) but moderate sensitivity (≈53–59%). Thus, low MNBI increases the likelihood of pathological acid exposure, whereas normal values do not exclude GERD. Clinically, MNBI can complement reflux indices in guiding management decisions, particularly in older children where its specificity is high. A low MNBI supports the likelihood of acid-related mucosal injury and may justify acid suppression or closer follow-up, whereas normal MNBI values in symptomatic patients could suggest functional or non-acid reflux mechanisms. Thus, MNBI offers an additional, noninvasive parameter to refine diagnosis and tailor treatment strategies in pediatric GERD.

Pediatric thresholds were notably higher than adult cut-offs (first cut-off 2292 Ω [33], then 1500 Ω [14]), underscoring the need for age-specific reference values.

Unlike prior studies reporting lower baseline impedance in infants [16], we found no significant inter-age differences. This likely reflects our methodology since MNBI was measured during stable nocturnal periods, minimizing artifacts from swallowing and feeding. Technical aspects (catheter size, channel positioning) and preserved mucosal integrity in infants with predominantly weakly acidic reflux may also explain these findings [31]. Variability across studies highlights the need for standardized pediatric MNBI protocols and multicenter normative data.

Physiologic differences, such as shorter esophagus, liquid feeds, and supine position, predispose infants to frequent weakly acidic reflux and lower baseline impedance [25,26,31]. With growth, improved clearance and posture stabilize impedance values.

Adult and mixed-age studies consistently report that MNBI is lower in patients with abnormal acid exposure and can complement symptom association and pH metrics by indexing mucosal integrity [14,24,29,34]. When interpreting MNBI results, caution is warranted in extrapolating adult-derived cut-offs to pediatric populations, given the developmental and physiological differences in esophageal function across ages. Pediatric evidence has been more limited, with heterogeneous methodologies and small cohorts [20,35]. Recent pediatric studies have begun to address this gap and provide reference data more relevant to younger patients. Cresi et al. proposed preliminary pediatric reference ranges for MII-pH parameters, highlighting lower baseline impedance in early life [36]. Similarly, Pop et al. demonstrated a strong correlation between conventional and simplified MNBI measurement techniques in children, supporting the reproducibility and clinical value of MNBI in pediatric GERD [30]. Moreover, Lorenzo et al. reported age- and condition-specific variations in MNBI among children with cerebral palsy, emphasizing the importance of pediatric-specific normative frameworks [37]. Our results align with these findings, reinforcing the need for pediatric-adapted MNBI interpretation rather than reliance on adult thresholds, and further underscore the necessity of large multicenter studies to define robust, age-specific reference values.

The observed laboratory differences primarily reflect age-dependent physiology rather than GERD-specific changes. Older children showed higher hemoglobin and IgA levels, consistent with established age-adjusted reference ranges [38,39], while infants had higher vitamin D concentrations, likely due to supplementation practices in early childhood. A non-significant trend toward higher IgE levels in older children probably reflects immune maturation rather than a reflux-related mechanism. The modest inverse correlation between weight percentile and gas reflux episodes may suggest that children with lower weight experience more aerophagia or reduced esophageal clearance, but this finding should be interpreted cautiously, as it may reflect developmental or nutritional influences rather than true pathological reflux [27,40,41].

This study contributes important pediatric-specific data on MNBI and reflux phenotypes. MNBI appears to be a robust, reproducible marker of mucosal integrity in older children and may complement standard RI in GERD evaluation. Future multicenter prospective studies are needed to define normative MNBI values across ages, validate cut-offs against clinical outcomes, and explore links between reflux, atopy, and motility. Integrating MNBI into routine reporting could enhance risk stratification and guide therapy, including acid suppression, dietary interventions, and allergy work-up where indicated.

### Study Limitations

The retrospective, single-center design introduces potential selection bias and limits generalizability. Pediatric GERD diagnostic thresholds remain less standardized than in adults, and normative MNBI data are scarce. In our study, The ROC-derived cut-offs were based on the present symptomatic cohort and therefore may not fully represent normative pediatric impedance values. Moreover, without healthy controls, pathological cut-offs cannot be firmly established. As this study included only symptomatic children referred for MII-pH evaluation, the findings primarily reflect a clinically selected population, limiting generalizability and preventing clear distinction between physiological and pathological reflux patterns in asymptomatic children.

Intra- and inter-observer reproducibility for MNBI measurements was not formally assessed in this study, which represents a limitation given the potential variability inherent to manual MNBI calculation, particularly in pediatric populations.

Future multicenter prospective studies are needed to validate pediatric MNBI reference values, refine age-specific cut-offs, and clarify its role in the clinical management of GERD and related conditions.

## 5. Conclusions

This study provides one of the most comprehensive pediatric evaluations of MNBI to date, demonstrating a clear inverse correlation between MNBI and acid exposure. MNBI proved clinically valuable in older children, where it reliably identified pathological reflux with high specificity, while its diagnostic utility in infants was limited. These findings highlight MNBI as a useful adjunct to pH-impedance monitoring for assessing mucosal integrity in pediatric GERD. Importantly, adult cut-offs are not applicable to children, underscoring the need for prospective multicenter studies to establish age-specific reference values and validate clinical thresholds.

## Figures and Tables

**Table 1:** Patient Demographics, Reflux Characteristics, and Laboratory Findings by Age Group.

Characteristics		Age		*p* Value
		<1 Year	≥1 Year	
Patients characteristics	Mean age	0.52 ± 0.03 years Range 0.08–0.95 years	7.18 ± 0.35 years Range 1.01–17.76 years	<0.0001
	Percentile weight	Median 22.24 (IQR: 4.25–38.99) Range 0.1–99	Median 22.33 (IQR: 4.66–56.25) Range 0.1–99.53	0.382
pH-detected episodes	Reflux index %	Median 3.15% (IQR: 1.2–8.75) Range 0.1–67.7	Median 2.45 (IQR: 1.1–5.12) Range 0–96.8	0.196
	Acid reflux episodes	Median 53 (IQR: 28.25–120.5 Range 8–513	Median 56 (IQR: 29.75–81.5) Range 0–631	0.552
	Long acid reflux episodes (>5 min)	Median 1 (IQR: 0–3) Range 0–29	Median 0 (IQR: 0–2) Range 0–39	0.197
	Boix-Ochoa Score	Median 17.9 (IQR: 12.03–41.13) Range 3.3–166.4	Median 16.45 (IQR: 8.92–30.33) Range 0.5–254.1	0.196
Impedance-detected episodes	Episodes detected by impedance	Median 62.5 (IQR: 42.5–92.75)	Median 46.5 (IQR: 26.75–67.25) Range 0–168	0.0002
	Acid episodes	Median 29 (IQR:14.25–49) Range 2–220	Median 25.5 (IQR: 12–45) Range 0–151	0.548
	Weakly acid episodes	Median 29 (IQR: 16–47.5) Range 5–125	Median 13.5 (IQR: 6.75–25) Range 0–96	<0.0001
	Non-acid episodes	Median 0 (IQR: 0–0) Range 0–1	Median 0 (IQR: 0–0) Range 0–13	>0.05
	Liquid episodes	Median 51.5 (IQR: 29.5–76.5) Range 5–227	Median 27.5 (IQR: 15–48) Range 0–151	<0.0001
	Mixed episodes	Median 7 (IQR: 3.25–12.75) Range 0–31	Median 9 (IQR: 4–14) Range 0–80	0.329
	Gas episodes	Median 32.5 (IQR: 24–49.75) Range 0–693	Median 49.5 (IQR: 24.75–94.25) Range 0–693	0.035
	Esophageal extension	Median 78.6% (IQR: 53.8–87.5%) Range 1.2–100	Median 69.5% (IQR: 49.5–80.63%) Range 0–100	0.013
Symptoms associated with reflux	Simptom index (≥50%)	45.16%	27.35%	0.999
	Symptom associated probability (≥95%)	57.14%	34.95%	0.031
MNBI	MNBI at channel Z6	4354 ± 259.9 Ω Range 1173–7457 Ω	4344 ± 167.8 Ω Range 729.3–10,000 Ω	0.740
	MNBI at channel Z5	4610 ± 247.8 Ω Range 1694–8710 Ω	4719 ± 159.6 Ω Range 811.3–10,000 Ω	0.993
Patients’ laboratory testing	Hb	11.51 ± 0.2 g/dL Range 9.8–13.8 g/dL	12.82 ± 0.09 g/dL Range 9.2–16.7 g/dL	<0.0001
	IgE	7.97 ± 1.37 U/mL Range 0.5–23.8 U/ml	136 ± 34.18 U/mL Range 0.26–3517 U/mL	0.106
	IgA	59.37 ± 25.4 U/mL Range 12.1–325 U/mL	113.6 ± 7.29 U/mL Range 19.7–270 U/mL	0.0007
	25-OH vitamin D levels	64.74 ± 7.79 ng/mL Range 50.1–83.7 ng/mL	27.31 ± 1.5 ng/mL Range 7.26–64.4 ng/mL	<0.0001

Abbreviations: MNBI, mean nocturnal baseline impedance; Hb, hemoglobin; IgE, immunoglobulin E; IgA, immunoglobulin A; 25-OH vitamin D, 25-hydroxy vitamin D; IQR, interquartile range.

## Data Availability

The research data can be provided upon request. The data are not publicly available due to privacy and ethical reasons.

## Abbreviations

The following abbreviations are used in this manuscript:

GERD	gastroesophageal reflux disease
MII-pH	Multichannel intraluminal impedance and pH
MNBI	mean nocturnal baseline impedance
IgA	Immunoglobulin A
IgE	Immunoglobulin E
RI	Reflux index
AET	Acid exposure time
SEM	Standard error of mean
IQR	Interquartile range
ROC	Receiver operating characteristic
AUC	Area under the curve
Hb	Hemoglobin
25-OH vitamin D	25-hydroxy vitamin D
